# Advancements and Future Directions of Dual-Target Chimeric Antigen Receptor T-Cell Therapy in Preclinical and Clinical Studies

**DOI:** 10.1155/jimr/5845167

**Published:** 2025-01-15

**Authors:** Chenyun Zhang, Haizhou Liu

**Affiliations:** ^1^School of Medicine, University of Tsinghua, Beijing, China; ^2^Division of Hematology-Oncology, Department of Medicine, University of Pittsburgh, Pittsburgh, Pennsylvania, USA

**Keywords:** cancer immunotherapy, dual-target CAR-T, hematological malignancies, solid tumors

## Abstract

In recent years, chimeric antigen receptor T-cell (CAR-T) therapy has made groundbreaking progress in the treatment of various cancer types, particularly hematological malignancies. In the meantime, various preclinical and clinical studies have extensively explored dual-target CAR-T therapies which can be designed to recognize two antigens simultaneously based on the immunophenotype of tumor cells. Compared with single-target CAR-T approach, dual-target CAR-T therapies demonstrate varying degrees of superior antitumor CAR effects, prevent antigen escape and relapse, reduce on-target off-tumor effects, and ensure durable responses in different types of cancer. These advantages highlight the potential future prospects in this field, showing varying degrees of advancement in preclinical and clinical studies. Herein, we aimed to review different dual-target CAR-T studies conducted on a wide range of tumor models, summarizing the selection of target combinations, the efficacy and safety demonstrated in preclinical and clinical settings, the existing limitations, and the potential future directions of this promising therapeutic strategy.

## 1. Introduction

Chimeric antigen receptor T-cell (CAR-T) therapy is a revolutionary immunotherapy used in cancer treatment, achieving groundbreaking progress in the treatment of various tumors, especially refractory/relapsed (R/R) hematological malignancies. This treatment involves collecting autologous T cells and transducing them ex vivo with an artificially modified receptor that combines the costimulatory domain of the T-cell receptor (TCR) with a single-chain variable fragment (scFv) derived from an antibody, often using a viral vector as the carrier [1, 2]. After lymphodepletion, these CAR-T cells are reinfused into the patient, where they can directly target and recognize specific tumor antigens without major histocompatibility complex mediation, thereby exerting efficient and highly specific tumor-killing effects. CAR-T cells have evolved over several generations to enhance their efficacy and stability. The widely used structure typically couples the cluster of differentiation (CD) 3-*ζ* domain with CD28 or 4-1BB costimulatory domains [3–8]; this configuration has demonstrated broad efficacy in preclinical and clinical studies across various disease models. CAR-T therapy has made notable progress in the treatment of B-cell tumors by targeting CD19, CD20, and CD22 antigens. In recent years, the Food and Drug Administration (FDA) has approved a series of related CAR-T therapies. Approximately 80% of patients achieve complete remission (CR) with early treatment, while ~50% of patients achieve 1-year event-free survival (EFS) with long-term treatment [8, 9]. Although the efficacy of CAR-T therapy has been widely validated, it still has significant limitations such as a high remission rate [10] and a high incidence of CAR-related side effects including cytokine release syndrome (CRS) [11], neurotoxicity, B-cell regeneration disorder, and high risk of lymphoma. These issues have been recently reported and emphasized by the FDA. In recent years, as more research has focused on examining the efficacy and safety of CAR-T therapy in clinical applications, the limitations of the widely used single-target CAR-T therapy have become more apparent.

### 1.1. Antigen Escape

Among the many limitations of the current CAR-T therapies, relapse due to antigen escape is a significant drawback. This occurs when CAR-T cells targeting a single antigen exert strong selective pressure on tumor cells, leading to the recurrence of tumors that do not express this antigen. Currently, multiple anti-CD19 CAR-T therapies for B-cell leukemia have resulted in relapses in ~30%–50% of patients with CD19-negative tumors, which negatively impacts prognosis [12–15]. Even CAR-T therapies targeting other antigens such as CD22 or B-cell maturation antigen (BCMA) cause relapses due to antigen loss, making this a common and significant obstacle to CAR-T therapy efficacy across multiple studies [16–18]. The downregulation or loss of CAR-T target antigens in tumor cells is a common mechanism of antigen escape relapse. In CAR-T therapy applied to multiple myeloma (MM), 4%–33% of patients experience relapse after receiving BCMA CAR-T therapy, potentially due to this mechanism [19, 20]. Moreover, aside from hematological malignancies, the efficacy of CAR-T therapy in solid tumors is further hindered by antigen escape relapse. Solid tumors have a more complex cellular composition and often exhibit heterogeneous antigen expression, making treatment even more challenging [21–23]. Some studies have also suggested that single-target CAR-T therapies have a weaker ability to cope with immune escape caused by the tumor microenvironment, often leading to suppressed efficacy [24].

In recent years, dual-target CAR-T cells have been successfully used in many preclinical and clinical trials to address tumor relapse caused by antigen escape from the original target. For example, combining two B-cell antigens, such as CD19, CD20, or CD22, has shown promising results [25–27]. Therefore, there is a growing belief that dual-target CAR-T therapy can overcome these limitations, and its efficacy can be enhanced.

### 1.2. On-Target Off-Tumor Effects

In addition to antigen escape, on-target off-tumor effects represent another significant obstacle encountered in the clinical application of single-target CAR-T therapy. Among CAR-T therapies targeting acute myeloid leukemia (AML), single-target CAR-T therapies against CD33 or CD123 have shown certain success [28, 29]. However, owing to the heterogeneity of AML blasts and antigen overlap between tumors and hematopoietic stem cells, CAR-T cells often fail to distinguish between tumor and normal cell lines, posing a significant challenge to the safety and efficacy of the therapy [30, 31].

### 1.3. Limitations of Efficacy

Another obstacle faced by single-target CAR-T cells is their relatively low efficiency. In certain diseases, such as refractory/relapse T-cell leukemia/lymphoma, tumor cells often exhibit significant heterogeneity. This makes it difficult for single-target CAR-T therapy to achieve sufficient efficacy, resulting in poor prognosis and even treatment failure [32, 33]. For example, single-target CD5 or CD30 CAR-T therapy used for the treatment of T-cell lymphoma (TCL) often requires extremely high doses to achieve the desired therapeutic effect [34]. High doses and repeated administration often result in higher costs and greater safety risks. Consequently, the application of CAR-T therapy in the clinical treatment of TCL remains limited. In this context, the use of bispecific CAR-T therapy may enhance the antitumor efficacy. Some studies on nanobody-derived bispecific CAR-T cells targeting CD13 and TIM3 in AML treatment have reported improved tumor suppression and reduced immunogenicity and toxicity [35].

### 1.4. Safety Concern

In recent years, among the various FDA-approved single-target CAR-T therapies, different degrees of CRS due to the long-term effects of CAR-T cells have been reported in multiple studies and are associated with poor prognosis [11]. Additionally, the FDA's 2023 announcement regarding the severe risk of T-cell malignancy associated with BCMA-directed or CD19-directed CAR-T immunotherapies has further raised concerns about the safety of existing single-target CAR-T therapies. Regarding efficacy control, current research primarily focuses on the development of nonviral CAR-T gene delivery platforms, such as mRNA-based, transposon-based, and nanoparticle-based approaches.

Additionally, some diseases are significantly affected by CAR-associated side effects, which greatly limits their application. In patients with primary central nervous system diffuse large B-cell lymphoma (PCNSL), CAR-T therapy is not routinely considered due to the risk of life-threatening cerebral edema caused by CRS and CAR-T-related encephalopathy syndrome (CRES) in tumors infiltrating the central nervous system [36]. It was only after a reported case where CD19-CAR T achieved CR that related CAR-T research, including the dual-target CAR-T approach, began to be explored in this disease context.

**Figure 1:**
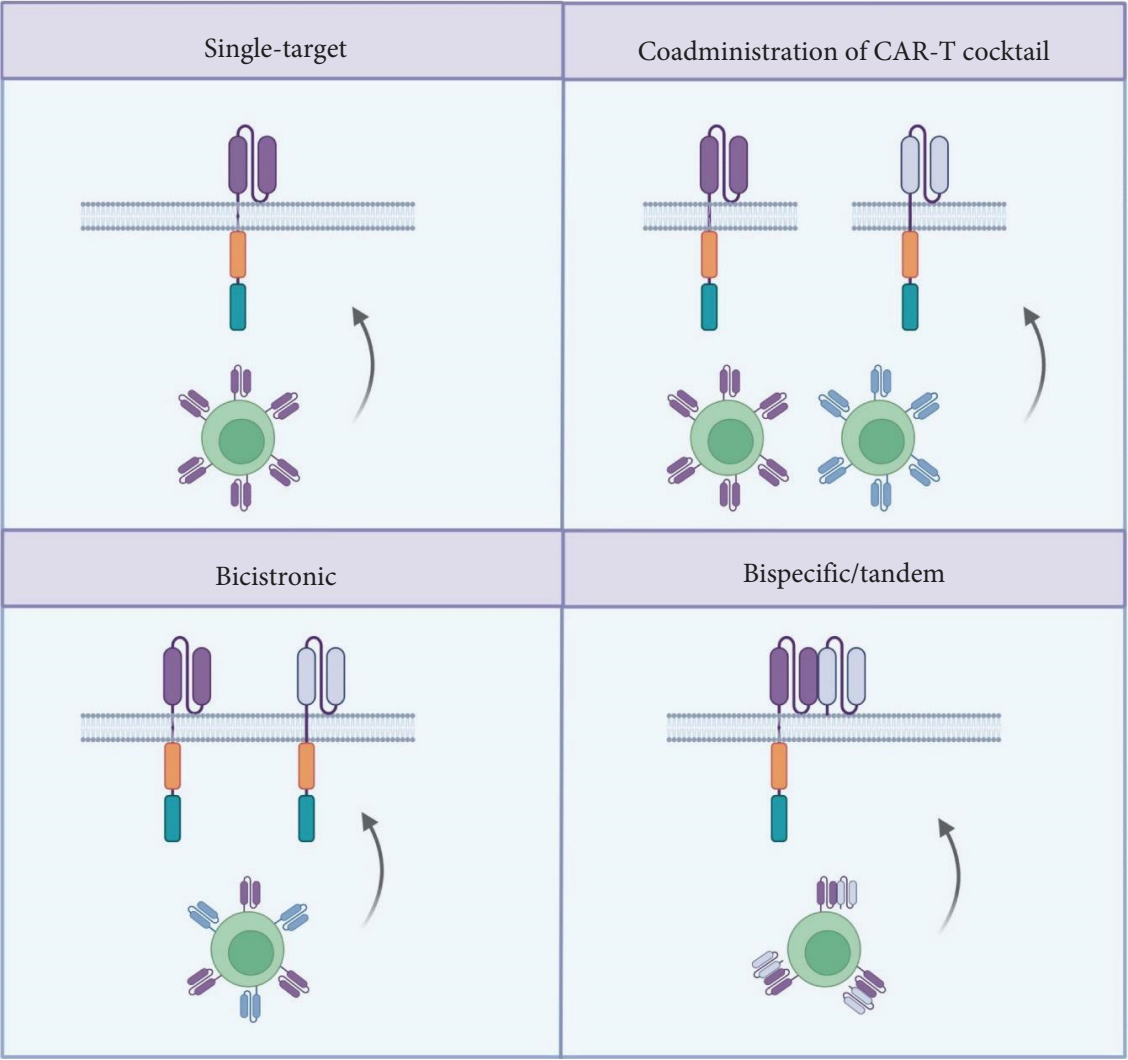
CAR-T cell targeting strategies. Single-target CAR-T: T cells express a CAR that recognizes a single antigen. Coadministration of CAR-T cocktail: A mixture of two single-target CAR-T cells, each recognizing different antigens, administered together. Bicistronic CAR-T: T cells express two separate CARs, each targeting a different antigen. Bispecific/tandem CAR-T: T cells express a single CAR capable of recognizing two different antigens simultaneously. Created in BioRender, https://BioRender.com/l43h222.

In response to pressing limitations, dual-target CAR-T therapy has emerged as a potential alternative to mitigate some of the drawbacks of single-target CAR-T therapy or to further enhance certain advantages. Targeting two antigens simultaneously can effectively suppress relapse, as tumor cells can be recognized by T cells as long as they express either one of the antigens, while the synergistic effect of recognizing multiple antigens may enhance efficacy and extend response durability [22, 37]. The selection of CAR-T therapy targets requires the consideration of multiple aspects, such as high coverage, specificity, efficacy, safety, and stability. Simultaneously achieving all of these criteria with a single target is nearly impossible [38]. Unlike single-target CAR-T cells, which only recognize one antigen, dual-target CAR-T cells can simultaneously recognize two tumor antigens. Several strategies can be employed to achieve this (Figure 1) [22, 37, 39]. Compared to single-target CAR-T, dual-target CAR-T can be achieved by constructing two separate single-target CAR-T cell populations and then coadministering this CAR-T cocktail into the patient's body. Another method involves using two CAR-encoding vectors to simultaneously transduce T cells, during which T cells are triggered to express two separate CAR proteins that target different antigens, thereby creating bicistronic CAR-T cells (T cells expressing only one CAR or lacking CAR expression entirely may also exist and can be isolated through cell sorting techniques). The final method involves combining two specific antigen-binding regions into one scFv to create a bispecific/tandem CAR that allows T cells to recognize two antigens with a single CAR. Several studies have employed different strategies to explore the feasibility and effectiveness of dual-target CAR-T therapies. However, the advantages and disadvantages of these strategies remain unclear. Building on its structure, the design of dual-target CARs typically follows three logical gates: “AND” (targeting cells that express both antigens), “OR” (targeting cells that express at least one of the antigens), and “NOT.” [40, 41] The previously mentioned CAR-T cocktail, bicistronic CAR-T, bispecific CAR-T, and AND-NOT SUPRA CAR [42] are examples of these practical applications. Recently, numerous studies have explored the performance of dual-target CAR-T therapy in various disease models and clinical settings. This review aimed to systematically describe recent advancements in dual-target CAR-T cell therapy in preclinical and clinical contexts, focusing on the design concepts, demonstrated efficacy and safety, limitations, and future development directions.

### 1.5. Rationale and Dual-Target Selection Strategy

Since the advent of the dual-target strategy in CAR-T therapy, numerous dual-target CAR-T antigen pair selections have been developed to treat various diseases. This approach is driven by the different antigen expression characteristics of tumor cells in various tumor models, disease features, and CAR-T adaptability. Each antigen pair was selected for different purposes and rationales (Table 1).

In various hematologic malignancies such as B-cell acute lymphoblastic leukemia (B-ALL), R/R non-Hodgkin lymphoma (R/R NHL), and R/R B-cell lymphoma, bispecific CAR-T cells targeting a second antigen in addition to CD19 (such as CD20 or CD22) have been extensively explored and applied in both preclinical and clinical settings to prevent antigen escape-related relapses [43–45, 47–49]. In addition to these two most commonly used secondary targets, other strategies that generate bispecific CAR-T cells combining CD19 with alternative antigens, such as CD79a or CD123 [44, 46], and CD37 [50] or CD38 [51] are also under further investigation.

In AML, where on-target off-tumor effects have been observed in single-target CD33 or CD123 CAR-T cell therapies, CD33-/CD123-bispecific CAR-T therapies have been designed to achieve more precise targeting [52]. CD123 coupled with CLL-1 bispecific CAR-T cells has also been explored with the aim of improving low response rates and extending survival times while simultaneously reducing off-tumor toxicity effects [53]. In TCL, which requires high-dose and repeated administration of CAR-T cells to achieve the desired therapeutic effect, leading to high costs and safety concerns, bispecific strategies combining existing targets, such as CD5 and CD7 [54] or nanobody-derived CD5/CD30 [55], present potential solutions to overcome these challenges.

In MM, in which BCMA is primarily selected as the CAR-T cell target, some studies have chosen CD19 as the second target to enhance CAR-T therapy efficacy and prevent relapse. A subset of stem cell-like myeloma cells reportedly expresses CD19, making it a viable target [56]. To date, studies have explored the feasibility of employing BCMA-CD19 CAR-T cells in treating MM through coadministration or bispecific targeting strategies [56, 57]. Additionally, FcRH5 and CD38 are considered potential secondary targets to overcome primary resistance and relapse in single-target CAR-T therapies [58–61]. For heterogeneous MM, which typically demonstrates stronger resistance to CAR-T therapies, selecting CS1 as a second target of dual-target CAR-T cells has garnered significant research interest, with clinical trials validating its potential [60, 62]. As the antigen expression profiles of tumor cells have been determined, other targets such as bispecific BCMA/TACI CAR-T cells have also been examined both in vivo and in vitro, demonstrating preliminary antitumor effects [63].

For solid tumors, which often exhibit poor CAR-T cell suitability owing to the unfavorable tumor microenvironment for T cells, limited T-cell infiltration, and heterogeneous tumor cell immune phenotypes, a bispecific CAR-T approach targeting epithelial cell adhesion molecule (EpCAM) and intercellular adhesion molecule 1 (ICAM-1) has been developed and explored with the aim of enhancing and sustaining the antitumor responses by addressing multiple challenges inherent to the treatment of solid tumors [64]. Additionally, bispecific CAR-T cells have shown varying degrees of progression in specific solid tumors. In ovarian cancer, bispecific CAR-T targeting PDL1 and MUC16 have been used to overcome on-target off-tumor toxicity [65]. In R/R PCNSL, a previous case report indicated that the tumor sample of a patient who experienced persistent relapse tested positive for both CD19 and CD70. As a result, fourth-generation CD19-CAR T cells and fourth-generation CD70-CAR T cells were assessed to determine their efficacy as potential treatment approaches [36]. In summary, as the concept of bispecific CAR-T cells was introduced in the field of cell therapy, various bispecific combinations targeting different tumors for different purposes have been widely explored in both research and clinical settings. Multiple factors are considered when selecting bispecific targets, including the antigen expression characteristics of the tumor, clinical response characteristics, and overall costs. Future exploration and refinement of bispecific CAR-T cells may offer breakthroughs in overcoming existing limitations.

### 1.6. Efficacy and Safety

#### 1.6.1. Preclinical

In the field of dual-targeting CAR-T cells, research on hematologic malignancies, particularly B-cell tumors, is undoubtedly the most extensive. In addition to CD19, the second target includes widely studied antigens, such as CD20, CD22, CD37, CD38, CD79a, and CD123.

A previous study using a CD19-loss lymphoma xenograft model found that CD19/CD79a dual-targeting CAR-T cells with different designs (tandem, bicistronic, and coadministration) were effective in preventing the recurrence of CD19-negative tumors at a relatively low required effective dose [46]. Compared with CD19 single-target CAR-T cells, both tandem and bicistronic designs demonstrated superior inhibition of JeKo-1 tumors; however, these designs showed a slightly reduced ability to recognize tumor cells positive for either CD19 or CD79a alone. Another study demonstrated that CD33/CD123 bispecific CAR-T cells achieved comparable in vivo efficacy in clearing AML as CD33 and CD123 single-target CAR-T cells. This underscores their ability to deliver precise therapeutic effects without inducing on-target or off-tumor toxicity, as bispecific CAR-T cells showed lower toxicity to CD34+ cells compared with single-target CAR-T cells [52]. In hematologic malignancies, where CAR-T therapy has been widely validated, or in more challenging applications such as solid tumors, bispecific CAR-T therapy with different targeted combinations has been thoroughly investigated at the preclinical stage. Most of these studies have demonstrated the efficacy and superiority of bispecific CAR-T treatments in vivo and in vitro as well as their potential resistance to antigen escape. However, the effectiveness and safety of bispecific CAR-T cell therapies in clinical applications require further validation using clinical trial data.

In a preclinical study targeting MM, FcRH5/BCMA bispecific CAR-T cells demonstrated superior tumor recognition and efficacy compared with single-target CAR-T cells [61]. In a subcutaneous luciferase-labeled NCI-H929 xenograft model, bispecific CAR-T cells significantly prolonged the survival time and enhanced tumor infiltration in mice compared with single-target counterparts. In another study targeting BCMA/CS1 as a dual target, dual-target CAR-T cells exhibited stronger antitumor capabilities compared with BCMA and CS1 single-target CAR-T cells [62]. Although CAR-T cell therapy alone has already achieved substantial tumor-free survival in mouse models and can be replicated in tumor rechallenge scenarios, combining it with anti-PD1 therapy may further accelerate tumor clearance. Additionally, the effect of different dual-target CAR designs on efficacy was investigated. Results showed that bispecific CARs were the most effective, while bicistronic and coadministration approaches had multiple drawbacks, including high costs, low transduction efficiency, and compromised efficacy.

In other hematological malignancies like TCL, Nb-derived bispecific CD5/CD30 CAR-T cells have been validated in both in vivo and in vitro experiments to demonstrate fratricide-resistant anti-T-cell cytotoxicity. The cells demonstrated significant tumor growth inhibition efficacy and potential for preventing relapse [54]. Another study investigated the efficacy of bispecific CD123/CLL-1 CAR-T cells in an in vitro AML model. For tumor cell lines with single-antigen positivity, the killing ability of the tandem CAR was comparable to that of the single-target CAR. However, for tumor cell lines with dual antigen positivity, the efficacy of tandem CAR was significantly superior to that of single-target CAR [53].

Studies on solid tumors revealed that although EpCAM CAR-T cells have the potential to clear tumors on their own, EpCAM/ICAM-1 bispecific CAR-T cells may overcome resistance and prevent relapse [64]. In both in vivo and in vitro models, bispecific CAR-T cells not only exhibited superior efficacy in tumors with homogeneous antigen expression but also demonstrated enhanced antitumor effects in tumors with heterogeneous antigen expression. In ovarian cancer, bispecific PDL1/MUC16 CAR-T cells demonstrated a significant therapeutic effect in an OVCAR-3 tumor mouse model [65]. Although no significant difference was found between the bispecific and single-target CAR-T cells in vitro, the bispecific CAR-T cells extended the survival time of mice by two to four times in vivo.

#### 1.6.2. Clinical

Currently, various dual-targeting CAR-T therapies targeting B-cell malignancies [25, 45, 47, 49, 66–72] and MM [56–59, 73–75] have reached the clinical trial stage, demonstrating varying degrees of efficacy and safety (Table 2).

A previous clinical trial targeting R/R NHL with CD19/CD20 bispecific CAR-T cells reported an overall response rate (ORR) of 90% and a complete response (CR) rate of 70% [47]; of note, one patient experienced relapse at 18 months but achieved CR after receiving the second dose of CAR-T cells. In terms of adverse reactions, none of the study patients developed neurotoxicity or CRS higher than grade 1, and only one patient experienced persistent cytopenia. This safety profile contrasts significantly with that of previous FDA-approved single-target CD19 CAR-T therapies, such as those used in the ZUMA-1 trial [76], in which 32% of patients experienced grade 3 or higher immune effector cell-associated neurotoxicity syndrome, and the JULIET trial [77], in which 23% of patients experienced grade 3 or higher CRS. In another clinical trial involving CD19/CD22 bispecific CAR-T cells for R/R B-cell lymphoma, the response rate was 87.5%, with a CR rate of 62.5% and a partial response (PR) rate of 25% [43]. Within 1-year post-treatment, three patients experienced relapse. During the trial, one patient developed grade 4 CRS, two patients developed low-grade CRS, and none developed neurotoxicity. In another study involving CD19/CD22 bispecific CAR-T cells for B-ALL, all six patients achieved MRD-negative CR without any observed neurotoxicity or grade ≥3 CRS but developed anemia [45]. One patient experienced relapse 5 months after treatment, with the relapsed blast cells lacking CD19 expression and showing reduced levels of CD22 expression [45]. Different perspectives were observed in a retrospective comparative study, which compared the efficacy of CD19, CD19/CD22, and CD19/CD123 single-target or dual-target CAR-T cells with donor lymphocyte infusion (DLI) [44]. The CAR-T group demonstrated significantly more favorable EFS than the DLI group, with a few number of patients developing acute graft-versus-host disease. However, no significant difference was found in the EFS and overall survival between single-target and bispecific CAR T cells. By contrast, in a previous case report examining the efficacy of CD19/CD70 bispecific CAR-T cells in R/R PCNSL, the patient's brain magnetic resonance imaging showed a CR [36]. During the 17-month follow-up period, the patient achieved disease-free survival without developing CRS, CAR-related encephalopathy syndrome, or other side effects.

Among the studies with extended follow-up time, one report stated that the OS and progression-free survival (PFS) rates for CD19/22 CAR-T therapy reached 77.3% and 40.2%, respectively [43]; meanwhile, another study observed that the experimental group (single-target or dual-target CAR) achieved an EFS of 516 days [44]. In a study analyzing a total of 219 patients with R/R B-ALL who participated in either CD19 (NCT03919240) or CD19/CD22 CAR T-cell therapy (NCT03614858) clinical trials, researchers found that patients receiving dual-target CAR-T therapy achieved a higher CR rate (98% vs. 83%) compared to those receiving single-target therapy [78]. Additionally, the incidence of adverse events was not significantly different between the two groups. Therefore, in this head-to-head comparison, dual-target CAR-T therapy demonstrated superior therapeutic efficacy compared to monospecific CAR-T therapy, where relapse significantly impacts PFS [12, 22]. Altogether these studies provide certain insight supporting the durable efficacy and long-term safety of dual-target CAR-T therapy.

In a phase 1 clinical trial of BCMA CAR-T therapy with or without CD19 CAR-T, dual-target CAR-T therapy through the coadministration of BCMA CAR and CD19 CAR did not show superior efficacy compared with BCMA CAR-T alone [56]. No significant difference was found in the median time to progression between the two groups (log rank *p*=0.4), partly because CD19 is not a relevant target in most patients, resulting in the limited antimyeloma potential of CD19 CAR-T therapy. Nonetheless, the safety of the coadministration of bispecific CAR-T cells was validated, as none of the patients developed high-grade CRS and only one patient experienced low-grade neurotoxicity. In another clinical trial using a bispecific strategy, the ORR reached 92%, with a median PFS of 19.7 months. Moreover, only 8% of the patients exhibited high-grade CRS [57]. However, hematologic toxicity remained a significant concern, as 100% of the patients experienced neutropenia and leukopenia.

In another clinical trial targeting BCMA and CD38, a high ORR was observed: 14 out of 16 patients showed a response (CR, 13, and PR, 1). However, owing to the limited number of cases, whether bispecific CAR-T therapy demonstrates superior efficacy remains uncertain [58]. In this clinical trial, different degrees of CRS were observed in 12 of the 16 patients (five patients with ≥grade 3 CRS). Another study demonstrated that BCMA/CD38 CAR-T cells exhibited stronger toxicity against heterogeneous MM in vitro compared with single-target CAR-T cells. In a subsequent clinical trial, 87% of patients responded to treatment, while 87% experienced varying degrees of CRS and common hematologic toxicities, such as neutropenia (96%) [59].

Bispecific CAR-T cell therapies have yielded diverse clinical outcomes in different disease models and clinical trials with various target selections. Most clinical trials have reported the ORR, CR, and relapse rates, as well as the common CAR-related side effects, including CRS, neurotoxicity, and hematologic toxicity. Hence, the superiority of bispecific CAR-T cell therapies in terms of overall cost and efficacy needs to be further evaluated; however, these clinical trial data undoubtedly support the potential for their continued application.

## 2. Future Directions

Despite the varying degrees of advantages achieved by dual-target CAR-T cell applications in different contexts, preclinical and clinical studies have observed various limitations and possible future directions.

In B-cell malignancies, several dual-target CAR-T therapies, such as CD19/CD20 and CD19/CD22, have already entered clinical stages, providing numerous insights and analyses from different perspectives. The challenges associated with the target selection of CD20 and CD22 have been widely discussed. First, the expression levels of CD22 in B-ALL and NHL vary, and CAR-T recognition often requires high expression levels [18, 79–82]. Additionally, the tandem configurations of CD19/CD22 scFvs have reduced binding affinity for each antigen [83]. Furthermore, the first-line treatment for NHL often includes monoclonal antibodies targeting CD20, which can create selective pressure, leading to heterogeneity or downregulation of CD20 expression in tumor cells [84, 85]. However, some trials have found that patients who underwent two or fewer chemotherapy sessions before receiving CD19/CD22 CAR-T therapy had better prognostic outcomes, and this consideration might be valuable for future treatment planning [43]. Although dual-target strategies often reduce the risk of antigen escape, further antigen loss and relapse have been reported in clinical trials. For instance, a CD19/CD22 CAR-T clinical trial reported a case of relapse with CD19 loss and downregulated CD22 expression. This finding suggests that further re-engineering of CAR specificity may be necessary to effectively prevent antigen escape [45]. Other studies examined the efficacy of different CAR-T structural designs. Compared with single-target CAR-T cells, tandem CAR-T cells exhibited reduced binding affinity for each antigen; in bicistronic CAR-T cells, the phosphorylation levels of downstream signaling pathways activated by CAR engagement were lower [46]. Therefore, designing alternative linkers or higher-affinity antigen-binding regions can potentially enhance the recognition capabilities of tandem CARs [86]. However, the large size of the constructs required for bispecific CAR-T cells and the potential involvement of switch-off systems further complicate viral vector packaging and transduction efficiency, potentially reducing the efficacy of CAR-T therapy [87, 88]. Additionally, when CAR-T cells express multiple scFvs, protein stability is compromised, leading to decreased affinity and specificity.

Hematological toxicity is a common occurrence in dual-target CAR-T therapy for MM. The occurrence of blood toxicity may be associated with certain factors such as lymphodepleting regimens and the optimized affinity of anti-CD38 CAR for hematopoietic progenitor cells [59]. However, the mechanism underlying the occurrence of cytopenia and the extent to which it affects patient prognosis remain unclear [57]. In several published studies, immune thrombocytopenia (ITP) has been reported following CAR-T therapy, highlighting the need for laboratory examination to enable specific and personalized evaluation of affected patients [60, 89]. Although different studies have presented varying viewpoints, some studies have indicated elevated levels of IL-6 and IL-10 in patients with ITP [90–92]. Although the specific mechanisms remain unclear, increased IL-6 and IL-10 levels may disrupt Treg/Th17 balance, activate CD8+ T cells, and stimulate antibody secretion, leading to immune-mediated platelet destruction.

In the successful application of EpCAM/ICAM-1 CAR-T against solid tumors, UBS54 CAR-T cells cause cytolytic toxicity to various primary cells with high EpCAM or ICAM-1 expression, such as epithelial cells from the human colon, kidney, and liver [64, 93]. The affinity of the UBS54 CAR might require further reduction and optimization to avoid on-target off-tumor effects. Other studies have focused on examining the controllability of CAR functions. For example, some researchers have suggested that modifying the structure of CARs can help avoid overstimulation and lymphopenia. One such modification includes the addition of safety switches, such as truncated human epidermal growth factor receptors [52]. As the application of dual-target CAR-T cell therapy in solid tumors remains in the exploratory stage, further investigation is required to determine suitable injection dosages, treatment cycles, and preclinical safety assessments [65].

When we look beyond clinical trials and focus on case reports from actual clinical applications, we find multiple approaches are being explored to integrate dual-target CAR-T therapy into real-world clinical settings. A case report described a patient with R/R MM who had undergone multiple rounds of chemo- and radiotherapy. Despite receiving BCMA/CS1 dual-target CAR-T therapy, the patient's tumor continued to progress, failing to suppress extramedullary relapse, while the poor prognosis was potentially associated with increased MYBL2 expression levels [94]. Another case report of a patient with Burkitt lymphoma, after failing multiple lines of chemotherapy, received donor-derived CD19/CD22 dual-targeted CAR-T cell therapy. The patient achieved partial PR and subsequently bridged to allogeneic hematopoietic stem cell transplantation (allo-HSCT), offering a new therapeutic possibility for patients with Burkitt lymphoma [95].

Overall, various studies have comprehensively reviewed the efficacy, safety, and other aspects of bispecific CAR-T therapy, highlighting current limitations across different stages of development. They also explore future avenues for research aimed at translating these therapies from preclinical to clinical settings.

## 3. Conclusion

To address issues related to antigen escape and relapse and to enhance precision therapeutic effects, various dual-target CAR-T approaches have been developed in recent years. Although dual-target CAR-T therapies have shown significant promise in preclinical and early clinical studies, their actual clinical application remains limited. These limitations include tumor cell heterogeneity, antigen downregulation associated with the second target, hematologic toxicities, and reduced affinity of tandem or bicistronic CARs toward individual antigens. Continued research efforts are crucial for refining and advancing dual-target CAR-T therapy. Future investigations should focus on enhancing specificity, improving delivery methods, optimizing dosing regimens, and expanding the scope of application to solid tumors. Addressing these challenges and harnessing the potential of dual-target CAR-T therapies holds substantial promise for improving the outcomes of cancer treatment.

## Figures and Tables

**Table 1:** Dual-target selection of CAR-T cells in published data.

Disease	Target	Rationale	References
ALL	CD19/CD22	Prevent antigen escape	[43–45]
	CD19/CD79a	Prevent antigen escape	[46]
	CD19/CD123	Prevent antigen escape	[44]

ALL/NHL	CD19/CD20	Prevent antigen escape	[47–49]

NHL	CD19/CD37	Prevent antigen escape	[50]
	CD19/CD38	Prevent antigen escape	[51]

AML	CD33/CD123	Overcome on-target off-tumor toxicity	[52]
	CD123/CLL-1	Overcome on-target off-tumor toxicity	[53]
		Improve low response rate	

TCL	CD5/CD7	Reduce cost and admistration dose	[54]
	CD5/CD30	Reduce cost and admistration dose	[55]

Multiple myeloma	BCMA/CD19	Overcome resistance and antigen escape	[56, 57]
	BCMA/CD38	Overcome resistance and antigen escape	[58–60]
	BCMA/FcRH5	Overcome resistance and antigen escape	[61]
	BCMA/CS1	Overcome resistance and antigen escape	[60, 62]
	BCMA/TACI	Prevent antigen escape	[63]

Solid tumor	EpCAM/ICAM-1	Enhance and sustain antitumor response	[64]

Ovarian cancer	PD1/MUC16	Overcome on-target off-tumor toxicity	[65]

PCNSL	CD19/CD70	Prevent relapase	[36]

Abbreviations: ALL, acute lymphoblastic leukemia; AML, acute myeloid lymphoma; BCMA, B-cell maturation antigen; CAR-T, chimeric antigen receptor T-cell; NHL, non-Hodgkin's lymphoma; PCNSL, primary central nervous system diffuse large B-cell lymphoma; TACI, transmembrane activator and CAML interactor; TCL, T-cell lymphoma.

**Table 2:** Published data of dual-target CAR-T therapy in clinical phase.

Disease	Target	Response	Adverse event	References
BCL, NHL	CD19/CD20	ORR: 79%–90% CR: 52%–74% PR: 7%–35% Relapse: 5%–14%	CRS: 50%–100% (0%–29% ≥ grade 3) CRES: 21%–27%	[25, 47, 49, 71, 72]

BCL, B-ALL, NHL	CD19/CD22	ORR: 72%–100% CR: 62.5%–100% Relapse: 15%–50%	CRS: 19%–100% (0%–85% ≥ grade 3) CRES: 0%–20%	[43, 66–70]

Multiple myeloma	BCMA/CD19	ORR: 92%–95% sCR: 43% PR: 14%	CRS: 60%–91% (0%–8% ≥ grade 3) Neurotoxicity: 0%–10% Neutropenia: 100%, Leukopenia: 100%, Anemia: 94%, Thrombocytopenia: 88%	[56, 57, 73]

Multiple myeloma	BCMA/CD38	ORR: 87%–88% sCR: 50%–81% PR: 18%–25% Median PFS: 9–17.2 months Relapse: 10%–21%	CRS: 75%–88% (25%–35% ≥ grade 3) Neutropenia: 96% Leukopenia: 87% Anemia: 43% Thrombocytopenia: 61%	[58, 59]

Multiple myeloma	BCMA/TACI	ORR: 43% PR: 29%	CRS: 45% CRES: 0%	[75]

Abbreviations: ALL, acute lymphoblastic leukemia; BCL, B-cell lymphoma; BCMA, B-cell maturation antigen; CAR-T, chimeric antigen receptor T-cell; CR, complete response; CRES, CAR-T-cell-related encephalopathy syndrome; CRS, cytokine release syndrome; NHL, non-Hodgkin's lymphoma; ORR, overall response rate; PFS, progression-free survival; PR, partial response; sCR, stringent complete response; TACI, transmembrane activator and CAML interactor.

## Data Availability

Data sharing is not applicable to this article as no new data were created or analyzed in this study.
